# Carbenoid Reactions Promoted by Solids: From Lewis to Brønsted Catalysts

**DOI:** 10.1021/acs.accounts.5c00159

**Published:** 2025-04-23

**Authors:** Antonio Leyva-Pérez, Marta Mon, Yongkun Zheng

**Affiliations:** Instituto de Tecnología Química, Universitat Politècnica de València, Agencia Estatal Consejo Superior de Investigaciones Científicas, Avda. de los Naranjos s/n, 46022 València, Spain

## Abstract

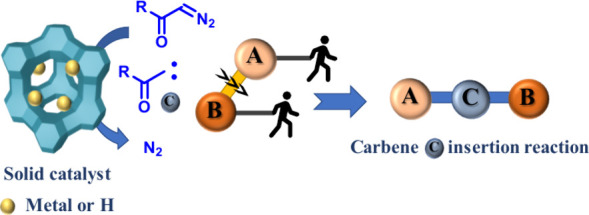

Diazocarbonyl compounds have become essential tools in organic synthesis, due to their ability to in situ generate reactive carbenes and be inserted in a variety of otherwise stable bonds, such as C–H, C–C, H–O, and so on. However, a soluble metal salt or complex catalyst is generally required to selectively activate and couple the carbene, and the metals employed so far are expensive (Rh, Au, Ag, Cu) and often unrecoverable. It is noteworthy that the price of ligands can make a cheaper metal catalyst (i.e., Cu) as expensive as other ligand-free noble metal catalysts. In the realm of modern sustainable chemistry, most of these methodologies are now unacceptable and must be adapted, and simple strategies for that include carbene photoactivation and the use of recoverable solid catalysts. Unfortunately, despite research in the field of carbene insertion reactions that has extended now for more than 50 years, examples with solid catalysts are still minor, and efficient solid catalysts have only been reported in the last two decades.

This Account shows the journey faced by our group in the last eight years to find solid catalysts for challenging carbene insertion reactions, employing diazocarbonyl compounds as carbene precursors. We will contextualize our results with those of previous solid catalysts. The discovery in 2017 that a quasi-linear Pd_4_ cluster stabilized within a metal–organic framework (MOF) was able to catalyze the Büchner and other carbene insertion reactions, spurred the design of supported metal clusters as catalysts for a variety of carbene insertion reactions. The Pd_4_-MOF could be reused 20 times in batch and implemented in a flow process. Following this, other catalytic solids, including Au and Ag as metals, not only in the same MOF but also on solid oxides and zeolites as supports, showed good activity for carbene insertion reactions and were also recoverable and reusable.

Our journey temporarily finishes in 2024 when “blank” experiments with a dealuminated zeolite surprisingly revealed that this simple solid acid, without any metal, easily activates the diazocarbonyl compound and catalyzes a variety of carbene insertion reactions, thus providing a cheap, commercially available, and reusable solid catalyst for these challenging reactions. Overall, rapid progress in solid-catalyzed diazocarbonyl compound activation, carbene formation, and insertion reactions has been achieved during these years, moving from expensive and difficult to prepare solid catalysts based on supported metal clusters to simple acid zeolites, pointing to confined Brønsted acids as the catalysts to study in the near future.

## Key References

- Fortea-PerezF. R.; MonM.; Ferrando-SoriaJ.; BoronatM.; Leyva-PerezA.; CormaA.; HerreraJ. M.; OsadchiiD.; GasconJ.; ArmentanoD.; PardoE.The MOF-Driven Synthesis of Supported Palladium Clusters with Catalytic Activity for Carbene-Mediated Chemistry. Nat. Mater.2017, 16, 760–76628604715 10.1038/nmat4910.^1^ This study showed that Pd, in the form of well-defined supported ultrasmall clusters, was able to catalyze some challenging carbene-mediated reactions from diazocarbonyl compounds and that the MOF solid was reusable and implementable in in-flow processes.
- Oliver-MeseguerJ.; BoronatM.; Vidal-MoyaA.; ConcepciónP.; Rivero-CrespoM. Á.; Leyva-PérezA.; CormaA.Generation and Reactivity of Electron-Rich Carbenes on the Surface of Catalytic Gold Nanoparticles. J. Am. Chem. Soc.2018, 140, 3215–321829460623 10.1021/jacs.7b13696.^2^ This work showed that supported Au nanoparticles, electronically enriched by virtue of the donor nature of the solid support, were able to catalyze some carbene-mediated reactions, without the molecular restrictions imposed by the microporous MOF.
- ZhengY.; Vidal-MoyaA.; Hernández-GarridoJ. C.; MonM.; Leyva-PérezA.Silver-Exchanged Zeolite Y Catalyzes a Selective Insertion of Carbenes into C–H and O–H Bonds. J. Am. Chem. Soc.2023, 145, 24736–2474537922487 10.1021/jacs.3c08317PMC10655197.^3^ This study showed that ultrasmall Ag species supported on zeolites catalyzed a variety of challenging carbene-mediated reactions from diazocarbonyl compounds and that the selectivity of the reaction depended on the counterbalancing cation of the recyclable solid zeolite.
- ZhengY.; EspinosaM.; MonM.; Leyva-PérezA.Dealuminated H–Y Zeolites Generate, Stabilize and Catalytically Insert Carbenes from Diazocarbonyl Compounds. J. Catal.2024, 440, 115835.^4^ This work showed that protons were able to catalyze a plethora of carbene-mediated reactions from diazocarbonyl compounds, when a commercially available and recyclable solid zeolite structure provides the suitable steric and electronic environment for the reaction to proceed.

## 1 Introduction: A Historical Perspective

### 1.1 Early Soluble Brønsted Acid Catalysts

Ethyl diazoacetate (EDA, **1a**) was synthesized by Theodor Curtius in 1883^5^ from readily available compounds such as glycine ethyl ester hydrochloride and nitrous acid, and since then, EDA **1a** and related diazocarbonyl compounds have become indispensable tools in organic synthesis.^6^ Despite their potential explosiveness, diazocarbonyl compounds can be prepared in high scale and have been used in industrial syntheses, since they combine relative handling and storage stability with unique reactiveness.^7^

**Figure 1:**
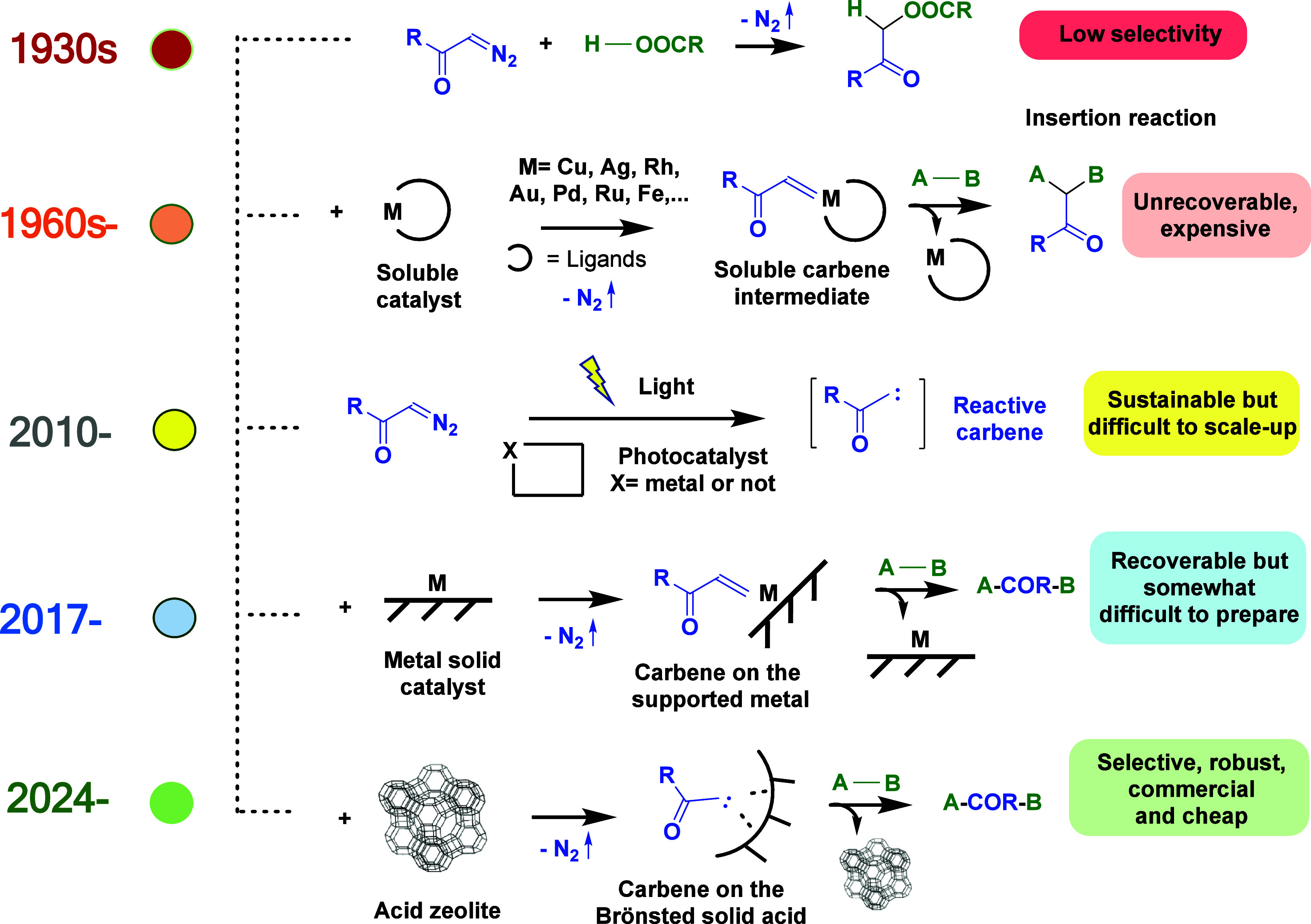
Proposed timeline of research on catalytic insertion reactions of diazocarbonyl compounds.

Figure 1 shows that the high reactivity of diazocarbonyl compounds comes from the easy formation of a carbene after N_2_ extrusion, triggered by either heating,^8^ light^9^ or, more conveniently, metal-catalyzed activation,^10^ thus giving a reactive carbene moiety ready to insert in a plethora of otherwise highly unreactive bonds (C–H, C–C, O–H, etc.).^11^

The formation of the carbene is unproductive and unselective when induced by just heating, which leads to different undesired by-products such as the hydrogenated carbene product, carbene dimers, or just decomposition products.^8^ This thermal decomposition, which in the case of EDA **1a** starts to occur at <150 °C, was recognized early by the chemistry community, and in 1931, the same Johannes N. Brønsted together with Ronald P. Bell used a variety of acids instead of heat to catalyze the insertion reaction of EDA **1a** in H_2_O or alcohols, in order to further prove the acid theory.^12^ Previously, in 1928, Robert Robinson and William Bradley reported the insertion of diazoketone in acetic acid,^13^ and both studies indicated a low catalytic efficiency of the acid. In any case, these seminal results in the field of catalytic activation of diazocarbonyl compounds, paradoxically settled the belief that protons (Brønsted acids) were not suitable catalysts for carbene generation and drove the research in the field toward metal catalysts for nearly a century, until only recently (in the past decade) an efficient catalytic activity by Brønsted acids has been addressed.

### 1.2 Soluble Metal Catalysts

**Figure 2:**
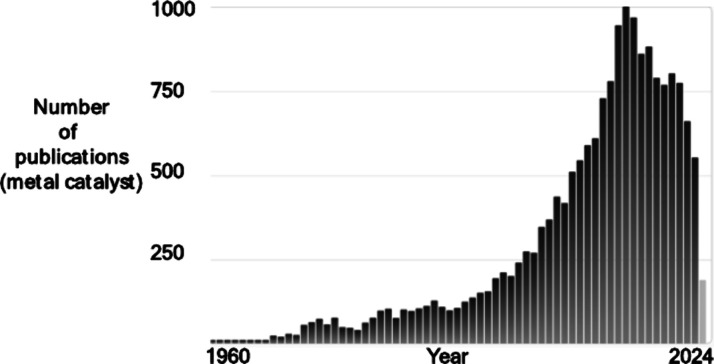
Number of publications on metal-catalyzed carbene insertion reactions from 1960 to 2023. Source: Scifinder.

Figure 2 shows the extraordinary increase in publications on metal-catalyzed carbene insertion reactions observed from 1960 to 2023 (carbenes as ligands in catalytic metal complexes are not considered).^14^ Hundreds of metal-catalyzed systems have been published, most of them with Rh,^15^ Cu,^16^ Ag,^17^ and Au^18^ catalysts, but also with Pd,^19^ Fe,^20^ and Ru.^21^ It is noteworthy to comment here that the related carbene cyclopropanation reaction is not considered in this Account not only for not being an insertion reaction within a broken single bond but also for having been deeply reviewed elsewhere.^22^

Rh_2_(OAc)_4_ can be considered the benchmark catalyst for the activation of diazocarbonyl compounds in solution.^23^ However, its rocketing price rapidly moved researchers to other metals such as Cu and Ag.^24^ These metals can also act as chiral catalysts with proper chiral ligands, constituting the main toolkit for carbene-mediated enantiomeric transformations.^25^ After the unveiling of Au as an extraordinary metal catalyst for organic reactions, Au catalysts were deeply studied from the 1990s to now, in many cases exceeding the catalytic activity of the parent Cu and Ag catalysts. Au is cheaper and less toxic than Rh; however, all the metal salts and complexes cited above were used as soluble catalysts during reaction, with an intrinsic lack of recoverability, leading to extremely expensive and toxic catalytic processes, even at the laboratory scale. In-flow processes have been designed in order to palliate all these issues;^26^ nevertheless, the necessity not only of a more economic but also of a more sustainable chemistry in the modern production methods, also at the laboratory scale, brought the necessity of designing solid catalysts to activate and react diazocarbonyl compounds (see Figure 1).

### 1.3 Photocatalysis

A direct photolysis of the carbene precursors, combined or not with photoredox or photosensitized catalysts, is a valuable strategy to trigger the desired carbene reactions.^27^ Although the photoactivation of diazo compounds has been known for decades, it has not been but in the last 15 years when efficient processes for carbene generation and engagement with other organic reactants have been achieved, many of them with the intermediacy of organic or metallic photocatalysts, somehow in analogy with the thermal activation.^28^ Besides, the metal-free photoprocesses are more cost-economical and sustainable than the metal-catalyzed counterparts, enabling clean process with minimal waste generation.^29,30^

### 1.4 Solid Metal Catalysts

Previous examples on solids to catalyze carbene insertion reactions have been mainly based on immobilized metal complexes, including Rh^31−33^ (some of them in the last 5 years),^34−37^ Ru,^38^ and Cu.^39,40^ In some cases, single atoms and controlled aggregated metal species are catalytically active,^41,42^ and some examples of metal-free solids can be found.^43^ The cyclopropanation reaction can be considered as the more studied reaction involving diazocarbonyl compounds and, as a consequence, solid metal catalysts were reported early for this reaction, more than 25 years ago;^44^ however, most of these catalytic systems were not extended to carbene insertion reactions, although some were with good success.^45−48^Figure 1 also shows our proposed timeline for the historical research on diazocarbonyl compounds as carbene precursors for insertion reactions, and it can be seen that solid catalysts have appeared only very recently.

### 1.5 A Resurgence of Brønsted Acid Catalysts, Now in Solid Form

The vision that protons (Brønsted acids) are unselective to catalyze the activation and insertion of diazocarbonyl compounds has somewhat changed in the last years after a plethora of studies with metal complex catalysts have unambiguously demonstrated that the chemical environment of the catalytic site is key for a proper formation and stabilization of the reactive carbene, thus leaving room to choose not only metals among a series of options in the periodic table, if the coordination sphere is adequate, but also perhaps protons. A prominent example of that is the recent Nobel-prize-winning directed evolution of enzymes, where metals in proteins can catalyze reactions with carbenes in biologically relevant environments.^49^ Therefore, Brønsted solid acids might be able to catalyze the insertion reaction of diazocarbonyl compounds if the chemical environment around the protons is suitable to that,^50^ as we will show here for zeolites.^4^

## 2 Results and Discussion

### 2.1 Electron-Rich Supported Metal Cluster Catalysts

**Figure 3:**
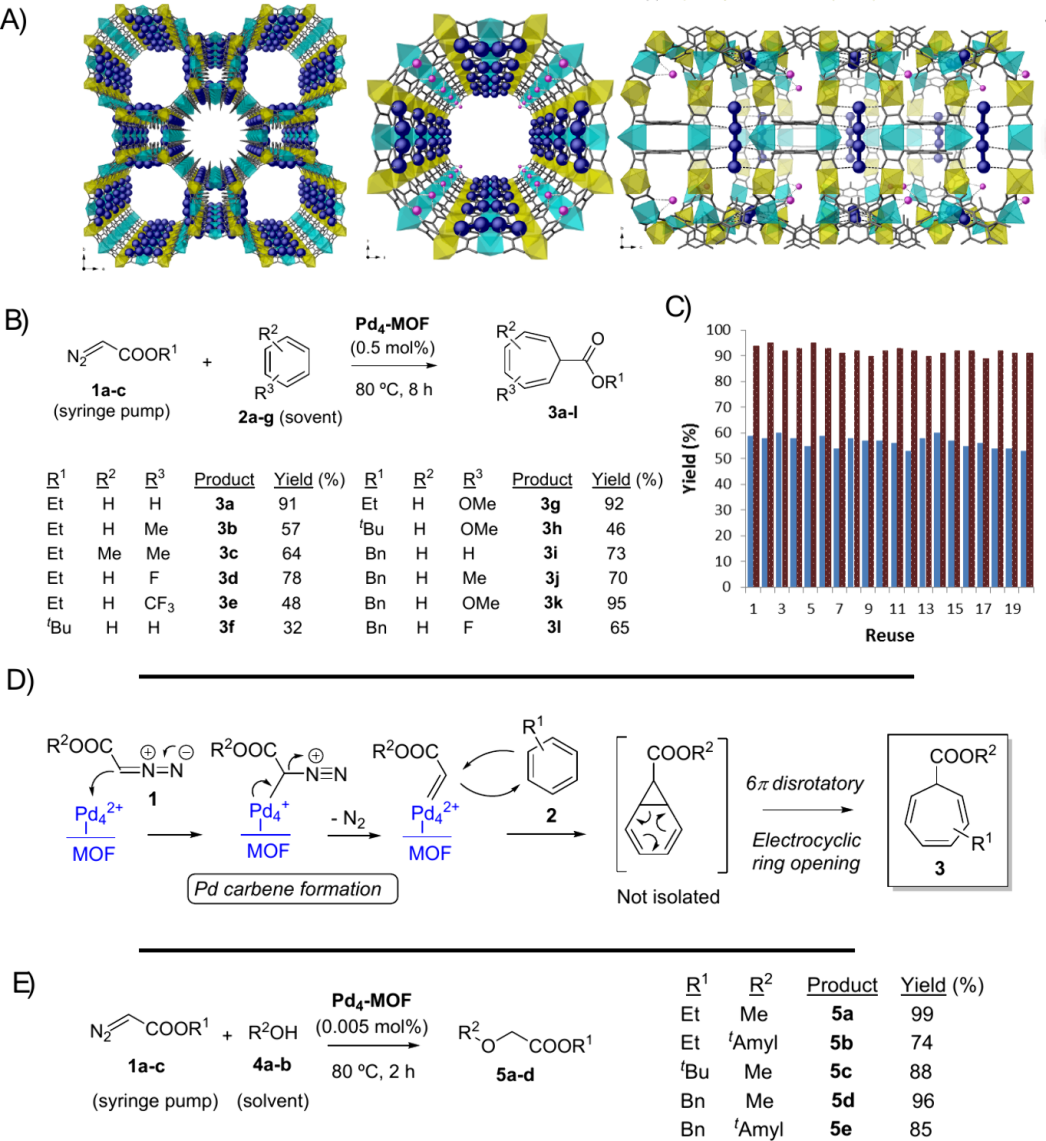
(A) Single-crystal X-ray structure of the Pd_4_-MOF shown under different perspective views. (B) Scope for the Pd_4_-MOF catalyzed intermolecular Buchner reaction. (C) Reuses of Pd_4_-MOF during the synthesis of product **3a**, either adding EDA **1a** at once (blue bars) or by syringe pump during the reaction time (red bars). (D) Plausible mechanism for the Pd_4_-MOF catalyzed intermolecular Buchner reaction of diazocarbonyl compounds **1** with aromatics **2**. (E) Scope for the Pd_4_-MOF catalyzed intermolecular carbene insertion reaction in alcohols. Figure adapted with permission from ref (1). Copyright 2017 Springer-Nature.

Metal clusters are metastable nanosized or sub-nanosized chemical structures where metal atoms are directly bonded between them, held by an external skeleton of either ligands or a structured framework (solid) to avoid decomposition.^51^ Our group has worked during the last years in the use of well-defined supported-on-solid metal clusters for catalytic organic processes^52−54^ and, in 2017, we reported that a Pd_4_-metal organic framework (Pd_4_-MOF) was able to catalyze the Buchner reaction of different diazocarbonyl compounds **1** with aromatics **2**.^1^ The crystalline structure of the material, resolved in a synchrotron beamline, is shown in Figure 3A and consists of a quasi-linear array of four Pd atoms coordinated to the MOF walls, together with interstitial individual Pd atoms. The MOF support is a highly porous anionic structure (virtual pore size ≈2 nm) of formula Mg^II^_2_{Mg^II^_4_[Cu^II^_2_(Me_3_mpba)_2_]_3_}·45H_2_O [Me_3_mpba^4–^ = *N*,*N*′-2,4,6-trimethyl-1,3-phenylenebis(oxamate)], very robust, and able to bear different postsynthetic processes. Indeed, the solid Pd_4_-MOF material was obtained after a three-step procedure consisting in two exchange transmetalation processes from the starting Mg^2+^-MOF, first to Ni^2+^-MOF and then to Pd^2+^-MOF, and a final reduction process of the accessible Pd^2+^ cations in the channel with NaBH_4_, to give the final Pd_4_-MOF. A total of 6 wt % of Pd in the MOF was achieved, and despite this high metal loading, the metal cluster structure remained well-defined after the synthetic process.

The Pd_4_ cluster holds a brut charge +2, as assessed by a combination of Fourier transform infrared spectroscopy (FT-IR) measurements under CO, in this case CO-probe diffuse reflectance infrared Fourier transform spectroscopy (DRIFTS), and X-ray photoelectron spectroscopy (XPS) measurements, together with density functional theory (DFT) calculations, among other techniques.^1^ The positive charge is essentially distributed among the corner atoms, leaving a simplified electronic structure for the Pd_4_ cluster of Pd^+^Pd^0^Pd^0^Pd^+^. Figure 3B shows that this highly reduced metal species is able to catalyze the Buchner reaction of a variety of different diazocarbonyl compounds **1a–c** and aromatics **2a–g** to give 12 different products, **3a–l**, in moderate to very good yields (32–91%). The steric hindrance around the diazo compound plays a role during the reaction, since ^*t*^Bu-substituted diazo compounds (compounds **3f** and **3h**) gave consistently the lowest yields (<50%) while the corresponding diazo compounds substituted with other alkyl groups (such as compound **3a**), including an electron-deficient group similar to ^*t*^Bu, in this case Bn (compound **3k**), gave >90% yield. This effect can be related to the steric restrictions imposed by the microporous solid catalyst surface. The reaction does not give any product at this temperature without the catalyst present. The solid catalyst could be recovered and reused up to 20 times, as shown in Figure 3C, or even operated in flow with solvent recycling during hours.

The reaction mechanism proceeds by a typical Lewis-acid catalyzed, back-donating activation reaction, as shown in Figure 3D, where the polarized Pd_4_ cluster activates the mildly nucleophilic diazo compounds **1** by virtue of the high electronic density of the metal cluster to liberate N_2_ and generate the carbene, ready to insert into the aromatic ring.^55^ A 6π disrotatory–electrocyclic ring opening process, typical of the highly unstable cyclohexadienyl-propyl bicyclic ring (norcaradiene), gives the final cycloheptatriene product **3**. Since the carbene formation on the supported metal cluster is the key step of the reaction, other bonds could also insert the carbene, and indeed, methanol **4a** and *tert*-amyl alcohol **4b** also showed good reactivity, to obtain the corresponding esters **5a–e** after O–H insertion in good to excellent yields (74–99%), as shown in Figure 3E. The catalytic amount of Pd_4_-MOF required for the O–H insertion reaction was very low (0.005 Pd mol %), and turnover numbers (TON) rounded 50000 were obtained in some cases. The Pd_4_-MOF material was, apparently, the only reported Pd catalyst efficient for a series of carbene insertion reactions from diazocarbonyl compounds, at that time.^1^

**Figure 4:**
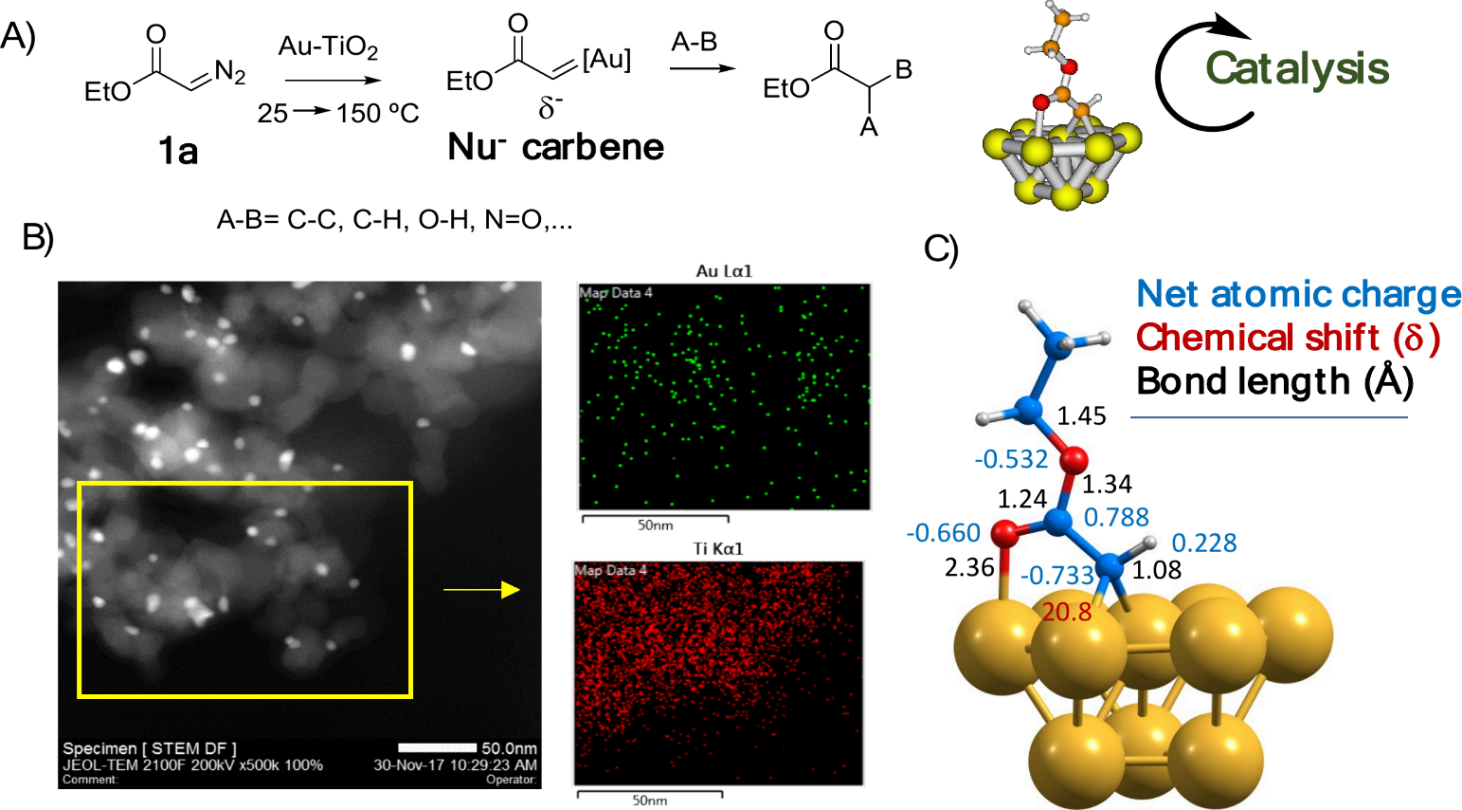
(A) Au-TiO_2_ catalyzed activation of EDA **1a** and insertion reaction in different bonds. (B) Representative HAADF-STEM image and EDX of Au-TiO_2_. (C) DFT calculations of the carbene on the Au NPs. Figure adapted from ref (2). Copyright 2018 American Chemical Society.

A high electron density on the metal cluster seemed to help the activation and insertion reaction of the diazocarbonyl compound. With this rationale in mind, the more electronegative metal in the periodic table, i.e., Au, could be catalytically active in the form of nanoparticles, thus maximizing the electron-richness of the Au atoms. It is worth commenting here that these Au nanoparticles would differ from catalytic Au complexes in solution for carbene insertion reactions where the Au atom acts as a Lewis acid, bearing a significant cationic charge. In the nanoparticle approach, the Au nanoparticle would act as an electron-rich metal site, balancing the number of Au atoms available for the catalysis, i.e., on surface, with the higher number of inner reduced Au atoms to gain electron density. The solid support could help to further gain electron density for the metal nanoparticle by providing electrons to the highly electronegative metal nanoparticle through their boundaries. Figure 4A shows this catalytic approach, with the formation of a nucleophilic Au carbenoid.^2^ Following the schematic representation of the diazo compound activation mechanism shown in Figure 3D above, the Au site would easily release N_2_ by back-bonding, forming the new carbenoid. For that, the Au nanoparticle must have a compromise between size and electron density, and TiO_2_ proved to be a proper support for this balanced Au nanoparticle nature. Other supports such as ZnO and CeO_2_ were also effective, and all of them present an open structure (not microporous), without substrate size restrictions, in contrast to the Pd_4_-MOF catalyst. Figure 4B shows a high-angle annular dark-field scanning transmission electron microscopy (HAADF-STEM) image of Au-TiO_2_, corresponding to ≈7 nm Au particle size on average, where the corresponding Au and Ti energy-dispersive X-ray spectroscopy (EDX) analysis, together with other techniques, confirmed the structure of the Au nanoparticle TiO_2_ support. Measurements of the Au-TiO_2_ catalyst by XPS, DRIFTS, Raman spectroscopy, and magic angle spinning ^13^C and ^15^N solid-state nuclear magnetic resonance (MAS ^13^C ss-NMR) with isotopically labeled samples of **1a**, and also DFT calculations, showed that the supported Au carbenoid presents nucleophilic character, with a Bader charge as negative as −0.73 on the metallocarbenoid atom, as shown in Figure 4C. The computed ^13^C NMR value for that carbenoid fitted the value observed by MAS ^13^C ss-NMR, completely distinct from a typical electrophilic metal carbenoid (≈200 ppm), and the calculated bond distances, CO-FTIR, and Raman values were in line with those experimentally observed.

The contiguous carbonyl group serves to stabilize the electron-rich carbene on the Au nanoparticle, allowing insertion reactions only with selected substrates. For instance, toluene, *n*-hexane, and ethanol were totally unreactive and could be used as a reaction solvent, in contrast not only to Au^+^ complexes but also to the Pd_4_-MOF catalyst.^1^ Indeed, the supported Au carbenoid preferred to be generated with electron-poor bonds such as C–H in cyclic α-diketones (diazodimedone) and the N=O bond of *ortho*-nitro phenylacetylenes. Noteworthy, substrates are relatively big and will hardly diffuse into the MOF structure of Figure 3A above. Typical reactions of classical metal carbenes such as Wolff rearrangements or alcohol insertion reactions were circumvented with the Au-TiO_2_ catalyst, and a Hammett plot confirmed the tendency of the Au-TiO_2_ carbenoid to better engage with electron-deficient molecules. The desired carbene reactions did not proceed in the absence of the Au-TiO_2_ catalyst.

**Figure 5:**
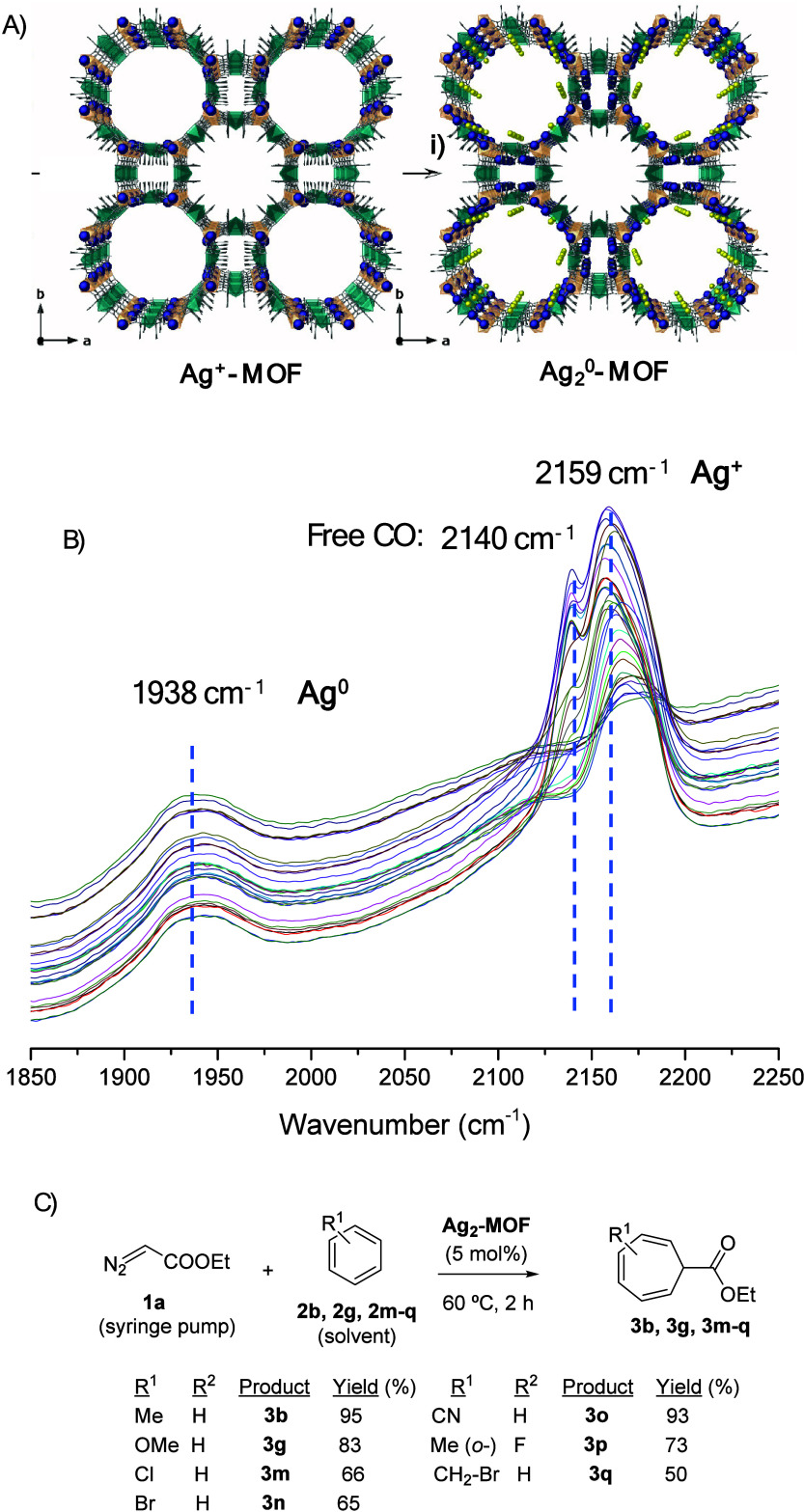
(A) Single-crystal X-ray diffraction structure of the Ag^+^-MOF (left) and Ag_2_^0^-MOF (right). (i) NaBH_4_ in MeOH at room temperature, with several additions until complete reduction. (B) DRIFTS of the Ag_2_^0^-MOF. (C) Catalytic results for the Buchner ring expansion reaction. Figure adapted from ref (57). Copyright 2022 American Chemical Society.

A further step in the use of reduced metal clusters as catalysts for the carbene formation and insertion reactions of diazocarbonyl compounds consisted of the use of supported Ag_2_ dimers. Ag is a cheaper metal than Pd and Au but, in contrast, is more difficult to stabilize as clusters of low atomicity.^56^ It was envisioned that the MOF used to generate and stabilize the challenging Pd_4_ clusters might also be helpful to prepare ultrasmall Ag clusters and, indeed, as shown in Figure 5A, the same post-synthetic strategy as with Pd enabled the synthesis of Ag_2_^0^ clusters.^57^

The three-step postsynthetic synthesis consisted, as for Pd_4_-MOF, in a first exchange of the Mg^2+^ by Ni^2+^ cations in the pores of the MOF, and then a second exchange by Ag^+^ ones, to yield the Ag^+^-MOF material which, after reduction with NaBH_4_, gave the Ag^0^_2_ clusters in the MOF (Ag_2_^0^-MOF). The total Ag wt % in the MOF was 11%. The single-crystal structures of both Ag-MOF materials were determined by X-ray diffraction in a synchrotron beamline and clearly revealed the formation of the Ag_2_ clusters, homogeneously distributed along the MOF channels. N_2_ adsorption isotherms for the Ag_2_^0^-MOF gave a calculated Brunauer–Emmett–Teller (BET) surface of 625 m^2^·g^–1^, and a DRIFTS study, shown in Figure 5B, shows three main peaks, one at 1938 cm^–1^, consistent with CO bridge-bonded to Ag^0^ atoms, a second peak at 2059 cm^–1^, attributable to Ag(CO)^+^ species, and a third peak at 2043 cm^–1^, corresponding to free CO, after saturation. Since the adsorption of CO on Ag^0^ is lower than on Ag^+^, the lower intensity of the former makes sense and could very well correspond to a 1:1 ratio between Ag oxidation states. These results strongly indicate the complete reduction of the accessible Ag^+^ cations to Ag_2_^0^ clusters in the MOF channels but not the reduction of the Ag atoms in the interstitial positions, which remained highly inaccessible and irrelevant for the catalysis as Ag^+^ cations.

Figure 5C shows the catalytic results for the Buchner reaction between EDA **1a** and different aromatics (used as both reactants and solvents). Good to excellent yields of products **3b**,**d**,**m**–**p** (65–95%) were obtained with 5 mol % Ag, covering a variety of substituents on the aromatic ring, including halogen, methoxy, and cyano functional groups. Please notice that 0.5 mol % Pd with Pd_4_-MOF was required for the same transformations (see Figure 3B above), but at higher reaction temperature (80 °C) and longer reaction times (8 h); thus the catalytic efficiency of both Ag and Pd MOFs can be considered similar. The starting MOF, without any metal clusters, catalyzed the reaction much more slowly, with an initial rate at least three times lower. The lower yield obtained with Ag_2_^0^-MOF for the bigger substrate **2q** (50%) could be attributed to the size discrimination exerted by the microporous MOF material, which was confirmed by the lack of reactivity of the bulkier aromatic 1,3,5-triisopropylbenzene and by the increase of the catalytic activity with the stirring rate (mass diffusion control). The fact that a measurable mass limitation effect is observed suggests that it is the solid and not the reaction itself that is imposing the steric hindrance, which connects with the ^*t*^Bu-diazoacetate results above in Figure 3B. A leaching test (hot filtration test at ∼30% conversion, at 60 °C) showed that the catalytically active species are on the solid within the experimental error (<10%), and in accordance with this result, the Ag_2_^0^-MOF could be reused up to five times without depletion in catalytic activity, after being recovered at the end of the reaction by centrifugation. The integrity of Ag_2_^0^-MOF after reaction was confirmed by powder X-ray diffraction (PXRD) and XPS, showcasing the ability of this MOF to stabilize otherwise fragile species such as the ligand-free Ag_2_^0^ clusters.

### 2.2 An Electron-Deficient Supported Metal Catalyst

Metal clusters can also be electron-deficient, particularly if generated under oxygen-rich environments. Given that ultrasmall Ag_2_ clusters were catalytically active for the Buchner reaction when embedded into a MOF (Ag_2_-MOF, see above), we wondered if ultrasmall Ag clusters could be generated within a zeolite framework and catalyze, perhaps in a different way, the carbene formation and insertion of diazocarbonyl compounds. In this way, we looked deeply into relatively cheaper metals as catalysts for carbene insertion reactions, avoiding Pd, Au, or Rh.

**Figure 6:**
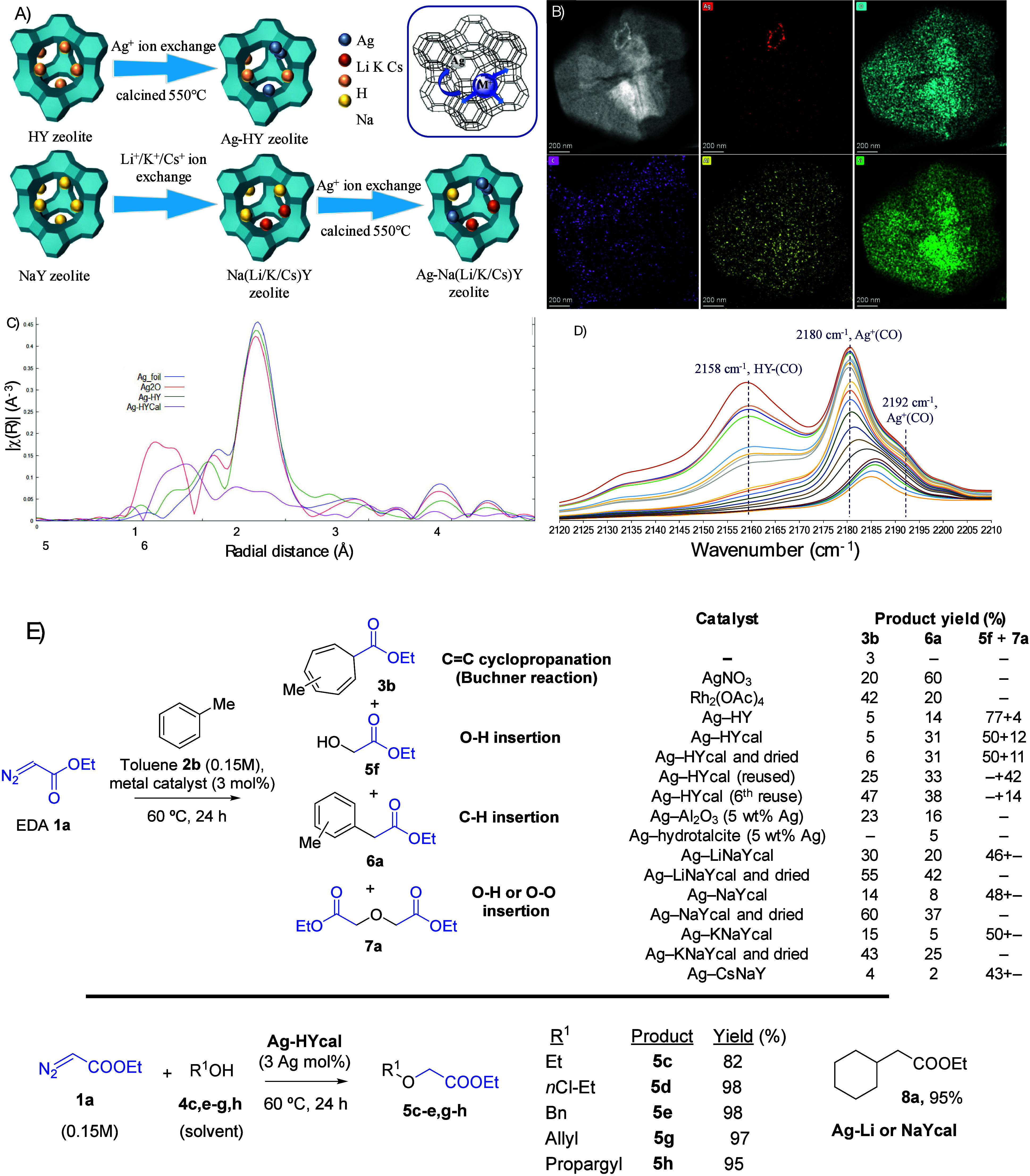
(A) Schematic representation of the Ag-zeolite synthesis.( B) (AC)HAADF-STEM image and EDX of the Ag-LiNaYcal crystallite. (C) EXAFS spectrum of Ag-HY (purple line). (D) DRIFTS of Ag-HYcal. (E) Catalytic results: the catalysts gave a mixture of products. Figure adapted from ref (3). Copyright 2023 American Chemical Society.

Zeolites are well-defined, crystalline, and microporous aluminosilicates with high inner surface, commercially available in both acid and base forms.^58^ The acid or base sites are mainly placed inside the zeolite cavities, thus isolated from each other, and generally associated with an exchangeable counterbalancing cation, which, in the case of the alkaline-earth group, can vary from H^+^ (acid zeolite) to Cs^+^ (basic zeolite). These exchangeable cations can be easily replaced by transition metal cations, in our case Ag^+^, which is isocationic to the alkaline-earth cations and facilitates the aqueous exchange. Figure 6A depicts the synthesis of the different Ag (1 wt %)-zeolitic materials, where the remaining counter-balancing cations on the zeolite framework control not only the electron density of the zeolite but also the steric hindrance in the channels and cavities, thus providing to variables to play with when designing the solid catalyst for the carbene reaction.^3^

Figure 6B shows an aberration corrected (AC) HAADF-STEM image of the prepared solids, in this case of Ag-LiNaYcal (“cal” means zeolite calcined at 550 °C under air after Ag^+^ exchange, see Figure 6 caption), combined with the EDX analysis, and it can be seen that Ag is homogeneously distributed across the zeolite framework (except some bigger bundles in the upper part of the image). Figure 6C shows the extended X-ray absorption fine structure (EXAFS) spectrum for another Ag-zeolite, in this case Ag-HYcal (purple line), compared with the uncalcined sample (green line) and Ag foil and Ag_2_O as standards (blue and red lines, respectively), and the results show that the Ag species are mainly ultrasmall Ag oxide clusters, perhaps together with Ag single atoms (compare the green and red lines). A DRIFTS study for the same Ag-HYcal zeolite is shown in Figure 6D, and the main bands appear at 2180 and 2192 cm^–1^, assignable to linearly coordinated Ag^+^(CO) and Ag^+^(CO)_2_, respectively, together with the expected band at 2158 cm^–1^ corresponding to the interaction between CO and the strong protons of the HY zeolite. Although some minor bands can be detected at lower wavenumbers (i.e., the band at 2133 cm^–1^), it can be clearly seen that the Ag clusters in the zeolite are cationic, in contrast to the Ag_2_^0^ clusters in the MOF, where the CO bands appeared at much lower wavenumbers (see Figure 5b above).^59^

Figure 6E shows the catalytic results for the insertion reaction of EDA **1a** added at once in toluene **2b** with different metals and the Ag-zeolites. The amount of catalytic Ag employed is 3 mol %, as in the Ag_2_^0^-MOF. Apart from the Buchner reaction, the insertion of the carbene into the methyl C–H bond of **2b** could occur, to give the corresponding benzyl ester **6a** (not significantly observed with the Ag_2_^0^-MOF catalyst). Not only that, the water strongly adsorbed in the zeolite might also react with **1a**, to give product **5f** after the O–H insertion reaction. Finally, product **7a** was also observed, coming from either a double insertion of **1a** in H_2_O or, alternatively, a O_2_ activation (see ahead). The results show that the reaction does not proceed without a catalyst under the indicated conditions (entry 1) but soluble AgNO_3_ gives 80% of the products (entry 2), mainly the C–H insertion product **6a**. The benchmark Rh_2_(OAc)_4_ catalyst for the Buchner reaction gives a 42% yield of Buchner product **3b** and 20% of C–H insertion product **6a**, plus dimers (35%, entry 3). Thus, it seems that the Ag zeolites and AgNO_3_ are more active and more selective that Rh_2_(OAc)_4_ under the present reaction conditions. Please notice that the C–C (Buchner) and C–H insertion reactions may follow different activation mechanisms; thus the catalytic activity of the Ag zeolite could change depending on the electronics or the sterics of the catalytic metal site, in turn controlled by the balancing countercation.^60,61^

The non-calcined Ag-HY zeolite shows complete conversion of **1a**; however, the main products found were neither **3b** nor **6a**, but **5f** and **7**, coming from the insertion of the carbene in the O–H bond of water present in the zeolite (entry 4). Only 19% of C–H-coupled products could be obtained. After calcination, Ag-HYcal showed a significant increase toward the C–C bond-forming coupled products **3b** (5%) and **6a** (31%); however, the main product still came from water insertion (62%, entry 5). This result indicates that the more strongly adsorbed water molecules in the Ag-exchanged HY zeolite are acting as a reactant during the carbene reaction. However, it could happen that the reuse of the zeolite would eliminate most of the O–H insertion products throughout the reuses; indeed, this is what happened, and the Ag-HYcal zeolite catalyst could be reused up to 7 times without depletion in catalytic activity (>90% conversion) and much better selectivity toward the C–C bond forming products **3b** and **6a** (up to >80% in uses 5–7, compare entries 7 and 8 in Figure 6E). Notice that the removal of chemisorbed water at 400 °C under vacuum overnight resulted in the zeolite color rapidly changing to brown, and a much lower conversion was found when it was used as a catalyst and the only products found were those coming from water, reflecting that some water was still there. In other words, attempts to thermally remove the chemisorbed water in the Ag zeolite only could lead to severe decomposition of the catalytically active Ag species. It is also noteworthy that the typical dimerization products for **1a**, i.e., diethyl fumarate and diethyl maleate, were not observed, since the isolated supported Ag sites avoid the encountering of two carbene fragments, which is an additional advantage of the supported Ag zeolite catalyst with respect to the MOF counterparts. For instance, the Rh_2_(OAc)_4_ catalyst gives significant amounts of these dimers (see entry 3).

Supported Ag nanoparticles (average size of ∼2 nm and 5 wt % Ag), on alumina (Al_2_O_3_) or hydrotalcite, were barely active for the reaction (entries 9 and 10), regardless of the support employed. In contrast, the change in the group I counterbalancing cation of the zeolite, from H^+^ to Cs^+^, led to significant changes in the catalytic activity. While the calcined cation-exchanged zeolite samples behave as Ag-HYcal, to give similar catalytic results (compare entries 5, 11, 13, and 15 in Figure 6E), if the Ag-zeolite catalyst is dried in situ before adding the reactants, the O–H insertion products **5f** and **7a** disappear and only the C–C coupling products **3b** and **6a** are formed, in yields up to 97% with Ag-LiNaY and Ag-NaY (entries 12 and 14, respectively). A hot filtration test for Ag-LiNaY showed that there is not any catalytically active species in solution, confirming the heterogeneous nature of the catalysis and the stability of the zeolite in reaction. In accordance, the PXRD and FTIR spectrum of the used Ag-LiNaY catalyst is similar to the fresh zeolite sample. Ag-CsNaY, which shows the highest amount of Ag^0^ species and cannot be dried at >100 °C prior to reaction since Ag spontaneously reduces, gives poor catalytic results (entry 17). The tendency of the Ag-HYcal catalyst to better activate the O–H bonds was confirmed when alcohols **4c–g** were used as solvents for the reaction. The results, also in Figure 6E, showed that the different alcohols engaged well with EDA **1a** under the same reaction conditions than toluene **2b** (60 °C, 24 h) to give the O–H insertion products **5c–g** in excellent GC yields (82–98%).

At this point, a highly challenging substrate such as cyclohexane was tested in view of the fact that a C–H insertion reaction occurred in the methyl group of toluene **2b**. In accordance to the results with **2b**, the Ag-HYcal was not active to react cyclohexane with EDA **1a**, and <30% of the insertion product **8a** was obtained, the rest being products **5f** and **7a**. In contrast, and in nice agreement with the results in Figure 6E, the Ag–Li or NaYcal catalysts gave the cyclohexane insertion product **8a** in 95% yield, under the same rection conditions than aromatics and alcohols coupled with EDA **1a**. These results, beyond showing a unique case of solid-catalyzed insertion reaction of a diazocarbonyl compound into an alkane, further confirmed the selective insertion of the carbene generated within the Ag zeolites into either C–H or O–H bonds as a function of the counterbalancing cations present in the zeolite, Li^+^(Na^+^) or H^+^ in this case, respectively.

### 2.3 A Neat Brønsted Acid Solid Catalyst

**Figure 7:**
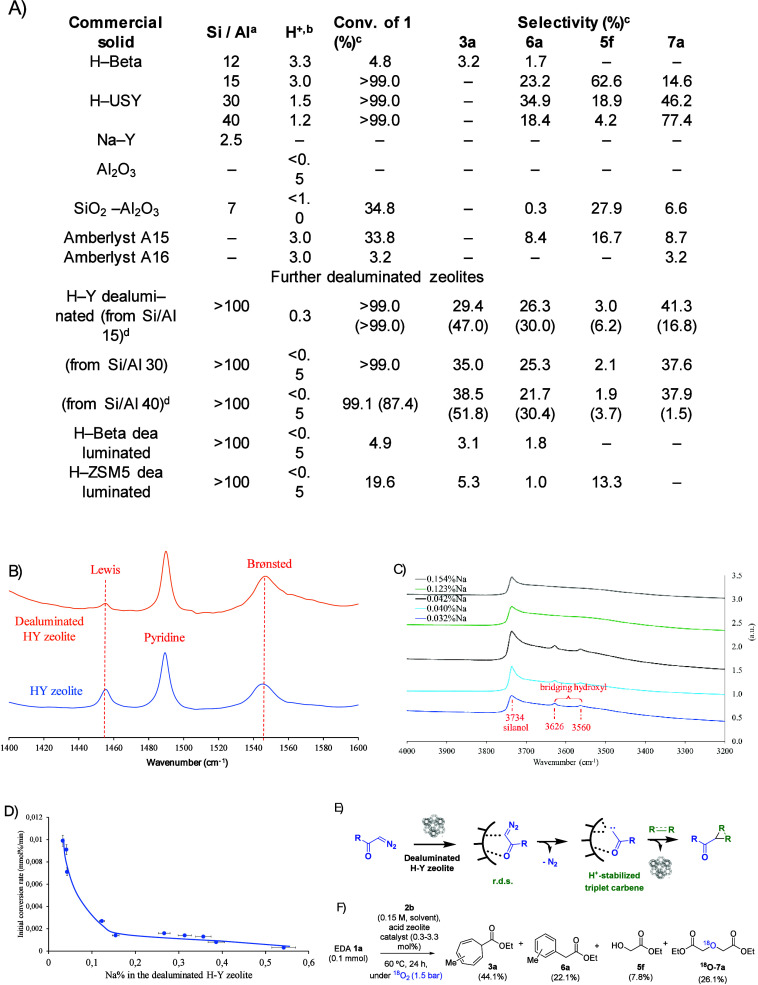
(A) Catalytic results for the reaction of EDA **1a** in toluene solvent **2b** (0.15 M) in the presence of different acid solids (30 mg, typically 0.3 to 3.3 H^+^ mol %) under the indicated reaction conditions. The catalysts gave a mixture of products. (B) Pyridine adsorption FT-IR bands for HY (Si/Al = 15) and dealuminated HY (Si/Al > 100) zeolites. (C) Diagnostic hydroxyl area in the FT-IR spectra of the Na^+^-exchanged zeolites after the evacuation of water under vacuum and heating. (D) Initial conversion rates vs different amounts of Na^+^ exchanged in the dealuminated HY zeolite (from Si/Al = 15) catalyst, for the reaction of EDA **1a** in toluene solvent **2b** (0.15 M) at 60 °C, for 24 h, and under air atmosphere. Error bars account for a 5% uncertainty. (E) Proposed mechanism for the generation, stabilization, and insertion of carbenes from diazocarbonyl compounds in catalytic dealuminated HY zeolites. (F) Results for the isotopic reactive experiment with ^18^O_2_. ^*a*^Provided by commercial houses and checked by inductively coupled plasma atomic emission spectroscopy (ICP-AES). ^*b*^Total number of Brønsted acid sites determined by pyridine titrations followed by Fourier-transformed infrared spectroscopy (FT-IR) after in situ adsorption/desorption cycles at different temperatures. ^*c*^Measured by gas-chromatography coupled to mass spectrometry (GC-MS) and checked by ^1^H, ^13^C and distortionless enhancement by polarization transfer (DEPT) nuclear magnetic resonance (NMR). ^*d*^Results between parentheses correspond to reactions performed under a N_2_ atmosphere. Figure adapted with permission from ref (4). Copyright 2024 Elsevier.

The blank experiment with neat HUSY zeolite during the above-mentioned studies on Ag zeolites revealed that the HUSY zeolite (USY: ultrastabilized Y zeolite) possesses minor but significant catalytic activity for the carbene insertion reaction in the O–H bonds, not in the C–C bonds, and that this catalytic activity of the zeolite only occurred for the acid form, while the neutral (NaY) and basic forms (KY or CsY) were completely inactive. Figure 7A show the results with EDA **1a** and toluene **2b** as coupling partners, performed under the same reaction conditions as for the Ag zeolite catalysts.^4^ It can be seen that, while the HBeta and NaY zeolites, and also alumina (Al_2_O_3_), did not activate EDA **1a** (entries 1, 5 and 6), not only different HY zeolites (entries 3–4) but also other Brønsted acid solid catalysts such as silicoalumina (SiO_2_–Al_2_O_3_, entry 7) or Amberlyst resins (entries 8 and 9) were catalytically active toward products **5f** and **7a**, pointing to H^+^ as potential catalysts for the activation and O–H insertion reaction of EDA **1a**.^4^

A decrease in the Si/Al ratio of the zeolite, which translates to a decrease of the extra-framework Al and of the total number of H^+^ sites in the zeolite (these H^+^ sites are nevertheless stronger when less Al is present in the zeolite framework), did not affect much the final yield of O–H insertion products (compare entries 2 to 4); thus further dealuminated zeolites were synthesized and tested as catalysts for the reaction of EDA **1a** with toluene **2b**.^4^ The results showed that, after further dealumination of the HY zeolite, the major insertion products were **3b** and **6a** instead than **5f** and **7a**; in other words, the insertion reaction switched toward the C–C and C–H insertion products. This unexpected reactivity did not occur for the dealuminated HBeta and HZSM-5 zeolites, which indicates unique selectivity of the dealuminated HY zeolite framework for the carbene generation of EDA **1a**. Indeed, the dealuminated HY zeolite was able to catalyze a plethora of insertion reactions of EDA **1a**, including not only aromatics and alkanes, but also alcohols, silanes and amines.^4,62^

Figure 7B shows the diagnostic area of a temperature-programmed FT-IR experiment on dealuminated HY zeolite with pyridine as a probe, and the results revealed that the Lewis acidity of the dealuminated zeolite sharply decreased after the dealumination process, as expected, while the Brønsted acidity remained constant. As commented above, the removal of the extra-framework Al does not change the overall Brønsted acidity of the zeolite, with stronger H^+^ sites. These strong H^+^ sites are mainly located in the bridging hydroxyl groups of the zeolite network, connecting two Si(Al) atoms (i.e., −Si–O(H)–Si−), and these bridging hydroxyl groups can be differentiated from the dangling silanol groups (−Si–OH) of the zeolite by the FT-IR technique. Figure 7C shows that the bridging hydroxyl groups were progressively removed from the zeolite after partial exchange with Na^+^, or what is the same, when the Brønsted acid zeolite was progressively converted into a neutral zeolite. Figure 7D shows that the initial rate for the reaction between EDA **1a** and toluene **2b** with catalytic amounts of these partially Na^+^-exchanged dealuminated HY zeolites decreases exponentially with the loss of bridging hydroxyl groups, which strongly suggests that two H^+^ sites participate in the reaction. Reactivity experiments with alkenes, and in situ FTIR and MAS SS ^13^C NMR measurements, indicated that a triplet carbene is formed from **1a**, which invoked a mechanism similar to catalytic metals for the formation of the carbene on the solid, as shown in Figure 7E, but in this case with two H^+^ sites providing the necessary empty orbitals to stabilize the triplet carbene.^4^

As commented above, the nature of formation of the ether product **7a** was still unclear, since a double O–H insertion of water in the carbene of EDA **1a** was unlikely (for instance, the O–H groups of the zeolite were apparently unreactive toward the O–H insertion reaction, as assessed by FT-IR analysis of the recovered zeolite catalyst). Further experimentation showed that product **7a** was formed in higher amounts in the presence of air, which may indicate that O_2_ participated during the process. Indeed, besides kinetic experiments, the demonstration that product **7a** came from an insertion reaction of the carbene in O_2_ and not from water was provided by the isotopically labeled experiment shown in Figure 7F, where ^18^O_2_ was employed as reactant, to give ^18^O-**7a** as the main *O*-insertion product.

## 3 Conclusions and Outlook

The activation, carbene generation, and insertion reactions of diazocarbonyl compounds, traditionally catalyzed by expensive and unrecoverable metal salts and complexes in solution,^63,64^ can experience a paradigmatic shift after employing well-defined solid catalysts. Initially starting with a metastable Pd_4_ cluster in a particular MOF (Pd_4_-MOF),^1^ different metal-supported catalytic solids for a variety of carbene insertion reactions have been discovered, including Au-TiO_2_,^2^ Ag_2_-MOF,^57^ and Ag in zeolites.^3^ These solid catalysts cover some of the more challenging carbene insertion reactions such as in C–C (Buchner reaction), C–H, O–H, and even O=O bonds, to name some of them. While the microporous materials (MOF and zeolites) are somewhat limited in the scope of molecules to functionalize by the size discrimination exerted by the pores, which in turn is the cause of the stability of the ultrasmall metal clusters, the inorganic oxides and the dealuminated zeolite supports, which present mesopores, are less restricted and enable reactivity of bigger molecules of interest for natural product synthesis.^65^ The discovery that a simple acid solid such as the dealuminated HY zeolite is able to catalyze a plethora of carbene insertion reactions with diazocarbonyl compounds^41^ will help to democratize these reactions, since an inexpensive and readily available solid catalyst is now reachable for the whole scientific community. Our results complement those previously reported in the literature with solid catalysts, since most of these previous catalysts were based on immobilized metal complex catalysts, which do not circumvent the synthesis of the metal complex and elaborated steps for the immobilization, while our catalysts here are based on robust ultrasmall metal aggregates.

We anticipate that future work on carbene insertion reactions with diazocarbonyl compounds will follow the principles of modern sustainable chemistry, with the design of new solid catalysts. We hope that the results in this Account will stimulate researchers, working not only in heterogeneous catalysis but also the same organometallic community, to pursue solid-catalyzed carbene reactions, where the stability, recoverability, environmental fingerprint, toxicity, and price of the catalyst will not be an issue and will not hamper the feasibility of the chemical reaction any more. For that, we propose here some potential lines of actions: (1) further studies on confined Brønsted acid catalysts, perhaps with other frameworks and even supramolecular hosts and enzymes; (2) use of the inexpensive and readily available first transition metals (Mn, Fe, Co, Ni, etc.), in cluster form preferentially,^66^ as catalysts for the carbene insertion reactions;^67−69^ (3) implementation of in-flow processes, much easier to design with solid catalysts than with soluble metal complexes; and (4) use of other precursors for carbenes as starting materials, such as α-carbonyl sulfoxonium ylides,^70^*N*-tosylhydrazones,^71^ or hypervalent iodine,^72,73^ which in some cases are in turn precursors for diazocarbonyl compounds,^71^ in order to improve the safety of the reactants for higher scale purposes. These suggestions might drive the discovery not only of new solid catalysts but also of new carbene insertion reactions.^74^
